# Validation of the Swedish Watson Dysphagia Scale for adult patients with eosinophilic esophagitis

**DOI:** 10.1093/dote/doab097

**Published:** 2022-01-22

**Authors:** Sofie Albinsson, Lisa Tuomi, Christine Wennerås, Helen Larsson

**Affiliations:** Department of Infectious Diseases, Institute of Biomedicine, Sahlgrenska Academy, University of Gothenburg, Gothenburg, Sweden; Department of Otorhinolaryngology, Head and Neck Surgery, Institute of Clinical Sciences, Sahlgrenska Academy, University of Gothenburg, Gothenburg, Sweden; Department of Otorhinolaryngology, Head and Neck Surgery, Sahlgrenska University Hospital, Gothenburg, Region Västra Götaland, Sweden; Department of Infectious Diseases, Institute of Biomedicine, Sahlgrenska Academy, University of Gothenburg, Gothenburg, Sweden; Department of Clinical Microbiology, Sahlgrenska University Hospital, Gothenburg, Region Västra Götaland, Sweden; Department of Otorhinolaryngology, Head and Neck Surgery, Institute of Clinical Sciences, Sahlgrenska Academy, University of Gothenburg, Gothenburg, Sweden; Department of Otorhinolaryngology, Head and Neck Surgery, NU Hospital Group, Trollhättan, Region Västra Götaland, Sweden

**Keywords:** eosinophilic esophagitis, patient-reported outcome measurement, Swedish, validation, Watson Dysphagia Scale

## Abstract

*Background:* The Swedish Watson Dysphagia Scale (S-WDS) has been used to assess dysphagia in patients with eosinophilic esophagitis (EoE) but has not been validated for this patient group. The aim of this study was to validate the S-WDS for adult patients with EoE. *Methods:* Ninety-seven Swedish adult patients with EoE and 97 controls without dysphagia filled out the S-WDS, the Swedish Eosinophilic Esophagitis Activity Index (S-EEsAI) and a set of supplementary questions. The reliability of the S-WDS was evaluated using Cronbach’s alpha, Pearson correlation of items and total score, and test–retest analysis (*n* = 29). Validity was investigated using Spearman correlations of the S-WDS items, S-EEsAI domains and a self-assessment score, and by investigating floor and ceiling effects. *Results:* The Cronbach’s alpha of the S-WDS was 0.77 and all items demonstrated moderate to strong correlation to the S-WDS score (*r* = 0.40–0.81) indicative of sufficient reliability of the instrument. In addition, the test–retest results reflected excellent reliability with an intraclass correlation coefficient of 0.85 for the S-WDS score. Adequate validity of the instrument was demonstrated, the S-WDS score correlated moderately with the self-assessment score and with 4/6 S-EEsAI domains, and strongly with the remaining two domains. Floor effects were more common for liquids and soft-textured foods and ceiling effects increased with increasing food consistency. The S-WDS scores of the patient group were significantly different from those of the nondysphagia control group (*P* < 0.001). *Conclusion:* The S-WDS instrument is an appropriate and valid instrument for assessment of dysphagia in patients with EoE.

## INTRODUCTION

Eosinophilic esophagitis (EoE) is a chronic inflammatory disorder of the esophagus. The eosinophil-predominant inflammation is induced by allergic responses to food- and/or inhalant allergens that are mediated by type 2 helper T (Th2) cells.^1^^,^^2^ The most frequent symptom of EoE in adults is dysphagia, other common symptoms include food bolus impaction, heartburn, and chest pain. Clinical manifestations of esophageal dysfunction in combination with histological findings of ≥15 eosinophils/high-power microscopic field (HPF) are required for diagnosis.^3^ Although the disease is chronic, symptom severity and inflammatory status of the esophageal mucosa fluctuate over time and can be alleviated by proton pump inhibitors, topical corticosteroids, and dietary restrictions.^3^

Patient-reported outcome (PRO) instruments are valuable tools for assessment of symptom burden, yet, there is only one Swedish PRO instrument validated for patients with EoE, the Swedish Eosinophilic Esophagitis Activity Index (S-EEsAI).^4^ Prior to the translation and validation of the S-EEsAI, other instruments, un-validated for EoE, were used for symptom evaluation. One of these instruments is the Swedish Watson Dysphagia Scale (S-WDS), which has been validated for assessment of dysphagia in patients with esophageal cancer.^5^ Although the S-WDS was not specifically developed nor validated for the assessment of dysphagia in EoE, the instrument has been appreciated among patients and has been used to monitor changes in symptom burden after treatment with topical corticosteroids in studies assessing dysphagia in patients with EoE.^6–8^ Moreover, items from the S-WDS were essential to provide stability in a multivariate model capable of separating histologic responders to treatment from nonresponders in patients with EoE who were treated with topical corticosteroids for 2 months.^9^ The S-WDS is user-friendly, takes a short time to fill out, is easy to use in the clinic, and is focused on the main symptom of EoE, dysphagia, which is why a validation of this established instrument for assessment of EoE is warranted.

The aim of this study was to validate the S-WDS for assessment of dysphagia in adult Swedish patients with EoE and consequently make two Swedish PRO instruments available to assess EoE symptoms.

## METHODS

### Respondents

Swedish adult patients with confirmed EoE (≥15 eosinophils/HPF in an esophageal biopsy and symptoms of esophageal dysfunction)^3^ were enrolled from the Ear, Nose, and Throat department at the NU Hospital Group, Trollhättan, Sweden. A minimum of 95 patients were sought, consistent with the recommendation regarding cohort size for validation according to Fayers and Machin.^10^ Inclusion criteria for the patients were ≥18 years of age, Swedish-speaking, and no additional diseases known to cause dysphagia. A control group consisting of individuals matched with the patient cohort based on sex and age, and without any history of esophageal diseases was recruited among acquaintances and colleagues of the authors. The respondents were enrolled via telephone and participation in the study was confirmed by written informed consent. The study was approved by the Regional Ethical Review Board of Gothenburg, Sweden (Dnr 2019-00602/1116-18).

### Study instruments

The S-WDS and the S-EEsAI instruments were distributed, along with supplementary questions concerning demographics and evaluation of the used instruments. Completion of the instruments was done either manually or electronically. A reminder was sent out by mail if the respondents had not filled out the instruments within a time span of 2 weeks. The questionnaires were sent once more to 29 randomly chosen patients for a test–retest analysis 2 weeks after the first completion of the instruments, i.e. a period during which no considerable change in symptom burden was expected to occur.

### Watson Dysphagia Scale

The Watson Dysphagia Scale (WDS) instrument is used to assess dysphagia brought on by a total of nine liquids and foods. It consists of nine items, all of which are answered on a 3-point Likert scale ranging from 0 to 1 (0 = never, 0.5 = sometimes, and 1 = always). The answer to each item is multiplied by a factor, which corresponds to the item number, i.e. item 1 (water) is multiplied by one, item 2 (milk) is multiplied by two, up to item 9 (meat), which is multiplied by nine. Each item score is summarized into a total score ranging from 0 (no dysphagia) to 45 (severe dysphagia). The layout of the instrument and the included items are presented in Table 1.

**Table 1:** Layout of the items in the Swedish Watson Dysphagia Scale

Item	Question
	Because of my swallowing difficulties, I have trouble to:
1	Drink water
2	Drink milk
3	Eat yoghurt
4	Eat jam or jelly
5	Eat omelet or mashed potatoes
6	Eat boiled vegetables, potatoes or fish
7	Eat soft white bread or pasta
8	Eat fresh fruit, e.g. apple
9	Eat meat, e.g. pork chop

The Swedish version of the WDS (S-WDS) was originally translated from English using the back-translation method^11^ and was validated for assessment of dysphagia due to malignancy of the esophagus.^5^ The S-WDS has not been validated specifically for EoE, but it has been used in multiple studies assessing dysphagia in Swedish patients with EoE.^6–9^

### Eosinophilic Esophagitis Activity Index

The Eosinophilic Esophagitis Activity Index (EEsAI) is a PRO instrument intended for assessment of symptom severity in adult patients with EoE, developed in 2014 by Schoepfer *et al*.^12^ The EEsAI takes into account both the dysphagia and behavioral adaptation related to EoE during a 7-day interval and consists of 10 items in five domains. The ‘visual dysphagia question’ (VDQ) domain concerns swallowing difficulties due to eight different food consistencies and is answered on a 0–3 scale (0 = no difficulties, 3 = severe difficulties). The ‘avoidance, modification, and slow eating score’ (AMS) domain is focused on the behavioral adaptation regarding the eight food consistencies and is answered using a yes/no format. The ‘frequency of trouble swallowing’ (Frequency) domain evaluates the occurrence of swallowing difficulties and is answered on a 0–3 scale (0 = never, 3 = daily troubles). In the ‘duration of trouble swallowing’ (Duration) domain the length of an episode of dysphagia is considered on a 0–4 scale (0 = no trouble, 4 = more than 5 minutes), and in the final domain, ‘pain when swallowing’ (Pain), the experience of pain when swallowing is answered as a yes/no question. The domain scores are summarized and transformed according to the EEsAI scoring manual into a total PRO-score, which ranges from 0 (no symptoms) to 100 (severe symptoms).^12^ Two additional questions that are not included in the PRO score are also part of the EEsAI instrument, these concern jaw injuries and explanation for any deviations in the VDQ domain.

The Swedish version of the EEsAI (S-EEsAI) has been validated and found suitable for evaluation of symptom severity in patients with EoE,^4^ and was included in this study as a comparison in the validity analysis of the S-WDS.

### Supplementary questions

Supplementary questions were supplied to the respondents in combination with the two instruments. The supplementary questions concerned demographics and feasibility of the instrument. Questions included how long it took to fill out the instruments, if help was needed in order to complete the instrument, and if any items were difficult or missing. For the patients with EoE, a supplementary question concerning self-assessment of disease severity ranging from 0 (no difficulties) to 10 (worst possible difficulties) was provided. For the nondysphagia control group an additional question regarding the incidence of swallowing difficulties and recent food impaction was provided.

### Statistical analyses

Statistical analyses were performed using the IBM SPSS Statistics software, version 26 (IBM, Armonk, NY, USA). A *P*-value of <0.05 was considered statistically significant. Ordinal data were presented as medians and range to provide a central tendency.

### Reliability and reproducibility

Evaluation of reliability was performed using the Cronbach’s alpha coefficient for the one scale that comprises the S-WDS (total score) and by Pearson correlation between each item and the total score. A Cronbach’s alpha value >0.70 was deemed as a satisfactory measurement of internal consistency.^10^ Pearson correlation coefficients ≥0.60 were considered as strong correlations, 0.40–0.59 as moderate correlations, and correlations ≤0.39 were considered as weak.^13^ Cronbach’s alpha and Pearson correlation with item exclusion was also performed, i.e. calculations of Cronbach’s alpha values without each item and correlation between the item and the total score with the item score removed from the total score.

The test–retest evaluation was assessed using intraclass correlation coefficient (ICC), which reflects the reproducibility of the instrument. Data from 29 patients who filled out the instruments twice during a 2-week interval was used to calculate an ICC value. Excellent reliability was reflected by ICC values >0.75, and moderate reliability by values of 0.40–0.75.^14^

### Validity

Construct validity was evaluated by Spearman correlations between the items and total score of the S-WDS and the domains of the S-EEsAI. Spearman correlation coefficients >0.70 were judged as strong, 0.30–0.70 as moderate, and <0.30 as weak correlations.^15^ Spearman correlations were also computed for the S-WDS total score and the self-assessment score of disease severity that was provided by the patients based on supplementary questions. We hypothesized that items 7–9 would demonstrate moderate correlations to the S-EEsAI domains VDQ, Frequency, Pain, and PRO score, and that items 1–6 would demonstrate weak correlations, but with a stepwise increase of strength starting with the weakest correlation for item 1. We also believed that a similar pattern regarding the correlation between the AMS domain and the S-WDS items would be seen albeit weaker. The Duration domain was hypothesized to demonstrate weak correlations with the S-WDS items, but again with increasing strength for increasing item number. Finally, we also hypothesized that the correlation between the S-WDS score and the self-assessment score would be of moderate strength.

Floor and ceiling effects for each item were determined. Floor effects were evaluated by determining the fraction of responses that provided the minimum score and ceiling effects by the fraction of maximum scores.^16^

Finally, the scores generated by the patient cohort were compared with the scores generated by the nondysphagia control cohort using two-tailed Mann–Whitney *U* test to evaluate the ability of the S-WDS to separate the two groups.

## RESULTS

### Study participants

About half of the eligible patients (*n* = 199) with EoE at the NU Hospital Group were included (*n* = 97) in the final study. The same number of nondysphagia control individuals (*n* = 97), matched for age and sex were also included. Approximately half of the patients (48%) were undergoing treatment for EoE during the study period, either in the form of proton pump inhibitors (*n* = 25), topical corticosteroids (*n* = 14), a combination of the two (*n* = 5), or dietary treatment (*n* = 2). One patient did not specify the type of treatment. The majority of the patients in the study were male (76%), reflecting the male predominance of EoE,^17^ and the mean age was 52 years. The nondysphagia control group also consisted of 76% males with a mean age of 52 years.

### Feasibility

Filling out the S-WDS, S-EEsAI, and supplementary questions was most frequently completed within 10–20 minutes for the patient cohort (63%) and in <10 minutes for the nondysphagia control group (67%). Both groups preferred the electronic version of the instruments over the paper version: 69% of the patient cohort and 91% of the nondysphagia control group used the electronic alternative. No patients reported that any of the questions were difficult to answer nor that any questions or information was missing from the S-WDS. There were no missing data for any of the items in the S-WDS among the study participants.

### Reliability and reproducibility

The S-WDS demonstrated good reliability and reproducibility as measured by Cronbach’s alpha, Pearson correlation and test–retest analysis. A Cronbach’s alpha of 0.77 was obtained for the S-WDS, which was judged to reflect satisfactory internal consistency. Analysis of Pearson correlations between the items and the total score of the S-WDS demonstrated that 5/9 correlations were strong and 4/9 were moderate (Table 2). Item exclusion revealed that Cronbach’s alpha was higher when item 1 was excluded, but not for any other items, and that all Pearson correlations were stronger when each respective item was included in the scale (Table 2). The test–retest analysis resulted in an ICC value of 0.85, indicative of excellent reliability.

**Table 2:** Reliability of Swedish Watson Dysphagia Scale items and total score estimated by using Cronbach’s alpha and Pearson correlation analyses

Item	Correlation with total score	Correlation with total score if item is excluded from score	Cronbach’s alpha if item is excluded
1	0.40^*^^*^	0.38^*^^*^	0.78
2	0.45^*^^*^	0.41^*^^*^	0.77
3	0.55^*^^*^	0.49^*^^*^	0.76
4	0.57^*^^*^	0.50^*^^*^	0.75
5	0.67^*^^*^	0.59^*^^*^	0.74
6	0.81^*^^*^	0.73^*^^*^	0.71
7	0.73^*^^*^	0.59^*^^*^	0.73
8	0.73^*^^*^	0.55^*^^*^	0.74
9	0.80^*^^*^	0.60^*^^*^	0.75

Weak correlation ≤0.39, moderate correlation 0.40–0.59, and strong correlation ≥0.60.

^^*^^*^^ *P* < 0.001.

### Validity

Spearman correlations between the items/total score of the S-WDS and domains/total score of the S-EEsAI were investigated to study the construct validity and are presented in Table 3. Weak correlations were demonstrated between S-WDS item 1–2 and all S-EEsAI domains/PRO score. Item 3 of the S-WDS only correlated moderately with the S-EEsAI PRO score and weakly with the remaining domains. Moderate correlations were frequently noted for items 4–9 of the S-WDS and the domains/PRO score of the S-EEsAI. The S-WDS score correlated moderately with all S-EEsAI domains, except for the VDQ domain and the PRO score, which demonstrated strong correlations.

**Table 3:** Spearman correlation between the Swedish Watson Dysphagia Scale items and total score, and the Swedish Eosinophilic Esophagitis Activity Index domains based on the results from the eosinophilic esophagitis patient cohort

		Swedish Eosinophilic Esophagitis Activity Index					
		VDQ	AMS	Frequency	Duration	Pain	PRO
Swedish Watson Dysphagia Scale	1	0.20	0.28^*^^*^	0.23^*^	0.19	0.17	0.28^*^^*^
	2	0.20	0.25^*^	0.27^*^^*^	0.12	0.14	0.25^*^
	3	0.25^*^	0.29^*^^*^	0.29^*^^*^	0.23^*^	0.26^*^	0.35^*^^*^
	4	0.28^*^^*^	0.30^*^^*^	0.38^*^^*^	0.30^*^^*^	0.23^*^	0.35^*^^*^
	5	0.45^*^^*^	0.40^*^^*^	0.39^*^^*^	0.32^*^^*^	0.27^*^^*^	0.48^*^^*^
	6	0.52^*^^*^	0.45^*^^*^	0.54^*^^*^	0.45^*^^*^	0.29^*^^*^	0.58^*^^*^
	7	0.51^*^^*^	0.49^*^^*^	0.46^*^^*^	0.40^*^^*^	0.30^*^^*^	0.56^*^^*^
	8	0.51^*^^*^	0.33^*^^*^	0.38^*^^*^	0.33^*^^*^	0.11	0.39^*^^*^
	9	0.69^*^^*^	0.63^*^^*^	0.61^*^^*^	0.53^*^^*^	0.21^*^	0.68^*^^*^
	Total score	0.72^*^^*^	0.61^*^^*^	0.65^*^^*^	0.56^*^^*^	0.30^*^^*^	0.71^*^^*^

AMS, avoidance, modification and slow eating score; Duration, duration of trouble swallowing; Frequency, frequency of trouble swallowing; Pain, pain when swallowing; PRO, patient-reported outcome score; VDQ, visual dysphagia question.

Weak correlation <0.30, moderate correlation 0.30–0.70, strong correlation >0.70.

^^*^^ *P* < 0.05.

^^*^^*^^ *P* < 0.001.

Spearman correlation between the self-assessment score and the S-WDS score demonstrated a moderate correlation of *r* = 0.59 (*P* < 0.001).

The distribution of scores was investigated (Table 4), including floor and ceiling effects. Floor effects, i.e. the overrepresented presence of minimal scores, were more common. Over 75% of the patients provided the minimum response for items 1–4, 50–75% gave the minimum response for items 5–6, and <50% of the patients provided the minimum response alternative for items 7–9 (Table 4). Ceiling effects were not as common, <10% of the patients provided the maximum response alternative for each of the items 1–7, and 12% and 25% of the patients gave the maximum alternative for items 8 and 9, respectively (Table 4).

**Table 4:** Eosinophilic esophagitis patients’ score distribution for the Swedish Watson Dysphagia Scale

Item	Number of levels	Median (min–max)	Floor *n* (%)	Ceiling *n* (%)
1	3	0 (0–1)	77 (79)	1 (1)
2	3	0 (0–1)	79 (81)	0 (0)
3	3	0 (0–3)	82 (85)	2 (2)
4	3	0 (0–4)	84 (87)	3 (3)
5	3	0 (0–5)	69 (71)	1 (1)
6	3	0 (0–6)	59 (61)	3 (3)
7	3	3.5 (0–7)	46 (47)	5 (5)
8	3	4 (0–8)	38 (39)	12 (12)
9	3	4.5 (0–9)	23 (24)	24 (25)
Total score	NA	12 (0–44)	14 (14)	0 (0)

NA: not applicable.

### Comparison between patients with EoE and nondysphagia controls

The S-WDS score and item scores for the patients with EoE were compared with the scores from the nondysphagia control group. All scores were statistically significantly different between the two groups with higher scores for the patient group throughout (Table 5).

**Table 5:** Swedish Watson Dysphagia Scale (S-WDS) results for patients with eosinophilic esophagitis and nondysphagia controls

S-WDS	Patients Median (min–max)	Nondysphagia control Median (min–max)	*P*-value^†^
1	0 (0–1)	0 (0–0)	<0.001
2	0 (0–1)	0 (0–0)	<0.001
3	0 (0–3)	0 (0–0)	<0.001
4	0 (0–4)	0 (0–0)	<0.001
5	0 (0–5)	0 (0–0)	<0.001
6	0 (0–6)	0 (0–3)	<0.001
7	3.5 (0–7)	0 (0–3.5)	<0.001
8	4 (0–8)	0 (0–4)	<0.001
9	4.5 (0–9)	0 (0–4.5)	<0.001
Total score	11.5 (0–43.5)	0 (0–7.5)	<0.001

^†^Mann–Whitney *U* test.

## DISCUSSION

To diagnose and assess the disease state of EoE, accurate interpretation of clinical symptoms is required, and the use of PRO instruments greatly facilitates this process. The S-WDS has been used in Swedish clinical practice and research to evaluate EoE, despite not being validated for the purpose.^6–9^ Through its repeated use, the S-WDS has been recognized as an appreciated and useful instrument for symptom evaluation for patients with EoE. This study therefore aimed to analyze the psychometric properties of the S-WDS.

The reliability and reproducibility of the S-WDS were deemed as good and valid throughout all analyses, only the comparison of the instrument’s Cronbach’s alpha value with a hypothetical Cronbach’s alpha value excluding item 1, difficulty drinking water, deviated from this pattern. However, this is not considered as a main symptom of EoE, although impaired esophageal function can result in difficulties in swallowing in general. Exclusion of item 1 from the scale would generate a slightly more reliable instrument for patients with EoE, however this difference was very minor. In addition, the maximum score of item 1 is 1, which has a marginal effect on the total S-WDS score of 45.

The S-WDS was compared with the S-EEsAI since the latter is an instrument developed specifically for the assessment of symptom severity in patients with EoE. This allowed us to evaluate how well the S-WDS reflected dysphagia related to EoE. Evaluation of validity using Spearman correlation analysis revealed that items 1–3 correlated with very few of the S-EEsAI domains and PRO score, indicative of them being inferior measurements of EoE-related dysphagia, compared with items 4–9, which correlated more strongly, just as hypothesized. Items 1–3 concern difficulties with drinking water, drinking milk, and eating yoghurt, neither of which are commonly associated with EoE.^18^ In contrast to our a priori hypothesis, the correlations between the S-EEsAI Pain domain and the S-WDS items were generally weak, probably because pain is not a primary symptom of EoE. Also rather surprisingly, item 8 of the S-WDS demonstrated overall weak correlations to the S-EEsAI domains. A possible reason for this is that item 8 concerns difficulties with eating ‘fresh fruit e.g. apple’, despite apple being listed as an example, fresh fruit could still be interpreted more broadly to include e.g. bananas, which are soft and untextured and generally not as difficult to ingest for patients with EoE.^18^ The moderate correlations of the S-WDS score with the S-EEsAI AMS, Frequency, Duration and Pain domains reflect that the S-WDS is only able to provide information on dysphagia and not regarding the patients’ behavioral adaptations, food modification habits, nor regarding the length or frequency of the episodes of swallowing difficulties. However, as the S-WDS scores strongly correlated with the S-EEsAI PRO scores we conclude that the S-WDS is a valid instrument for assessment of EoE-related dysphagia. This was further corroborated by the moderate correlation noted between S-WDS score and the self-assessment score of disease severity provided by the patients.

The presence of floor effects could be considered a limitation of this study and of the instrument. For items 1–6, more than half of the patients provided floor responses, which may raise doubts as to whether these items are relevant for the assessment of EoE. However, the inclusion of these items could lead to the discovery of other difficulties not commonly associated with EoE in individual patients. The floor effects may result from the instrument layout as there are only three scoring options per item, but also to the fluctuating nature of the disease,^19^ and ongoing medical treatment to alleviate symptoms.^20^ The ceiling effects became more frequent with increasing food texture, culminating with item 9, meat. Highly textured foods are more difficult to consume for patients with EoE,^18^ which the ceiling effects reflect.

Scores between patients and nondysphagia controls were statistically significantly different, indicating that the instrument can discriminate patients with EoE from nondysphagia controls.

The S-WDS was not originally developed for EoE, which was reflected by the poor correlations between item 1–3 and the EoE-specific instrument S-EEsAI, and by the increased Cronbach’s alpha value by exclusion of item 1. However, the S-WDS score is the measurement used for assessing dysphagia, not individual items, and the resulting S-WDS scores demonstrated very good reliability and validity for patients with EoE. Patient feedback and the lack of missing data confirmed that the S-WDS is a user-friendly questionnaire that complements the S-EEsAI. The S-WDS is adapted for clinical use with a compact format, consistent layout, and easy interpretation of the results whereas the S-EEsAI may be more adapted for research purposes. The implementation of a second Swedish PRO instrument validated for assessment of EoE will consequently increase the possibility of correct interpretation of disease severity and manifestation of clinical symptoms, and benefit both research and Swedish health care.

In summary, the S-WDS has been judged as a reliable and valid instrument for assessment of EoE-related dysphagia in Swedish adults with EoE.
